# Chronic Kidney Disease and Upper Tract Urothelial Carcinomas

**DOI:** 10.1155/2014/158918

**Published:** 2014-09-11

**Authors:** Li-Jen Wang, Joëlle L. Nortier, Bin Tean Teh, Cheng-Keng Chuang, Shen-Yang Lee

**Affiliations:** ^1^Department of Medical Imaging and Intervention, Linkou Chang Gung Memorial Hospital, 5 Fu-Hsing Street, Gueishan, Taoyuan 33333, Taiwan; ^2^Department of Medical Imaging and Radiological Sciences, College of Medicine, Chang Gung University, Taoyuan 33333, Taiwan; ^3^Institute of Radiological Research, College of Medicine, Chang Gung University, Taoyuan 33333, Taiwan; ^4^Department of Nephrology, Erasme Hospital, 1070 Brussels, Belgium; ^5^National Cancer Center and Duke-NUS Graduate Medical School, Singapore 169610; ^6^Department of Medicine, College of Medicine, Chang Gung University, Taoyuan 33333, Taiwan; ^7^Department of Urology of Linkou Chang Gung Memorial Hospital, Taoyuan 33333, Taiwan; ^8^Department of Nephrology, Linkou Chang Gung Memorial Hospital, Taoyuan 33333, Taiwan

Upper tract urothelial carcinomas (UTUC) have high prevalence rates in patients with chronic kidney disease (CKD), including dialysis patients and kidney transplant recipients. Analgesic nephropathy, Balkan endemic nephropathy, and aristolochic acid (AA) nephropathy share the common association with the development of CKD and UTUC. Genome studies allow identification of patients with genetic predisposition to Balkan endemic nephropathy and AA nephropathy. Furthermore, the identification of aristolactam deoxyribonucleic acid (DNA) adducts now provides robust evidence for AA exposure in UTUC patients, even in the absence of recallable exposure history. Diagnosis and treatment of UTUC in CKD patients are challenging. Clinical diagnosis of UTUCs in CKD patients with the available urological and imaging methods is much more difficult than in patients with normal renal function due to atrophic kidneys and poor excretory functions in these patients. High prevalence rates of contralateral upper tract and urinary bladder involvements of UC in CKD, either at presentation or recurrence, raise concerns of the necessity and techniques as well as perioperative risk of complete urinary tract exenteration. The standard treatment of radical nephroureterectomy with bladder cuff excision for UTUC patients may result in deterioration of renal function, rendering adjuvant chemotherapy unsuitable. This special issue collectively addresses these important topics regarding UTUC in CKD patients.

The authors in one paper nominate three mutant genes (CELA1, HSPG2, and KCNK5) in Balkan endemic nephropathy patients, which are associated with angiogenesis. This finding suggests that angiogenesis might play an important role in the development of Balkan endemic nephropathy. Another paper shows an elevated risk of urothelial carcinomas (UC) in ESRD patients with the analysis of a national wide cohort in Taiwan. Female patients have 9–18 folds of increased risk of UC as compared to 4–14 folds in male ESRD patients, which may reflect female predominance in AA consumption. One paper discusses the challenges of UTUC diagnosis in CKD patients. It reviews the detection rate of UTUCs in dialysis patients and kidney transplant recipients with the use of a variety of imaging and urological methods in the literature. Combination use of urological and imaging methods has been suggested for diagnosing UTUCs in symptomatic dialysis patients and kidney transplant recipients.

Another paper develops a predictive model for postoperative renal insufficiency in UTUC patients undergoing radical nephroureterectomy, which help in selecting eligible patients for cisplatin based adjuvant chemotherapy. Older age, lower estimated glomerular filtration rate before surgery, smaller tumor size, renal pelvis tumor, and absence of hydronephrosis or multifocal tumors are predictors for ineligibility for adjuvant chemotherapy. One paper describes modified incision complete urinary tract exenteration for UC in dialysis patients, providing short operative time, early oral feeding, and no major perioperative complications of this modified surgical procedure. Another paper reviews epidemiological evidence of AA exposure associated with occurrence of nephropathy and UC, including AA exposure scenarios in Belgium, Taiwan, and the Danube River as well as occupational exposure in Chinese herbalists. The identification of aristolactam DNA adducts in these patients further corroborates biological plausibility of AA exposure contributing to the occurrence of UTUC.

*Li-Jen  Wang* *Joëlle L. Nortier* *Bin  Tean  Teh* *Cheng-Keng  Chuang* *Shen-Yang  Lee*

